# Association between short-term blood pressure variability and target organ damage in non-dialysis patients with chronic kidney disease

**DOI:** 10.1186/s12882-024-03541-x

**Published:** 2024-03-21

**Authors:** Zhaoting Chen, Xinying Jiang, Jingcan Wu, Lin Lin, Zhengping Zhou, Man Li, Cheng Wang

**Affiliations:** 1 Division of Nephrology, Department of Medicine, The Fifth Affiliated Hospital Sun Yat-Sen University, 52 Meihua East Road, Zhuhai, Guangdong 519000 China; 2 Guangdong Provincial Key Laboratory of Biomedical Imaging, The Fifth Affiliated Hospital Sun Yat-Sen University, 52 Meihua East Road, Zhuhai, Guangdong 519000 China; 3 Department of Nephrology, Institute of Nephrology, The Second Affiliated Hospital of Hainan Medical University, 368 Yehai Avenue, Haikou, Hainan 570311 China

**Keywords:** Ambulatory blood pressure monitoring, Blood pressure variability, Weighted standard deviation, Chronic kidney disease, Target organ damage

## Abstract

**Background:**

It is unclear whether short-term blood pressure variability (BPV) is associated with target organ damage in patients with non-dialysis chronic kidney disease (CKD).

**Methods:**

A cross-sectional, single-center study was conducted among 3442 non-dialysis CKD patients hospitalized in the department of Nephrology of the Fifth Affiliated Hospital of Sun Yat-sen University from November 2017 to July 2022 and collected the demographic, laboratory, clinic blood pressure, ambulatory blood pressure data, and short-term BPV assessed by the weighted standard deviation (wSD) derived from ambulatory blood pressure monitoring (ABPM). Multivariate logistic analyses were used to evaluate the independent effects between short-term BPV and subclinical target organ damage, including left ventricular hypertrophy (LVH), abnormal carotid intima-media thickness (CIMT), low estimated glomerular filtration rate (eGFR), and albuminuria.

**Results:**

The average age of the participants was 47.53 ± 14.06 years and 56% of participants were male. The baseline eGFR was 69 mL/min/1.73 m^2^. Based on the tertile distribution of wSD according to equal numbers, patients were divided into three categories with T1(< 9.66 mmHg), T2(9.66–12.23 mmHg), and T3(> 12.23 mmHg) of SBPV; T1(< 8.17 mmHg), T2(8.17–9.93 mmHg), and T3(> 9.93 mmHg) of DBPV. The participants with the higher wSD group had a higher prevalence of target organ damage than their counterparts (*P*-trend < 0.05). An increasing trend in short-term variability was present with advancing CKD stages (*P*-trend < 0.001). Multivariate logistic analyses results showed that the odds ratio (OR) of SBP wSD was (1.07 [1.03,1.11], *P* < 0.001) for LVH, (1.04 [1.01,1.07, *P* = 0.029) for abnormal CIMT, (1.05 [1.02,1.08], *P* = 0.002) for low eGFR, and (1.06 [1.02,1.09], *P* = 0.002) for albuminuria; The OR of DBP wSD was (1.07 [1.02,1.12], *P* = 0.005) for LVH, (1.05 [1.01,1.09], *P* = 0.028) for abnormal CIMT, (1.05 [1.01,1.09], *P* = 0.022) for low eGFR, and (1.05 [1.01,1.10], *P* = 0*.*025) for albuminuria when adjusted for confounding factors and mean BP.

**Conclusions:**

In conclusion, short-term BPV is associated with target organ damage, and irresponsible of average blood pressure levels, in Chinese non-dialysis CKD participants.

**Supplementary Information:**

The online version contains supplementary material available at 10.1186/s12882-024-03541-x.

## Background

The prevalence rates of chronic kidney disease (CKD) worldwide have increased appreciably, seriously affecting human health [1–4]. Studies have revealed that cardiovascular events are the most leading reasons of death for people who suffer from CKD [5]. In addition to being a public health problem, hypertension have something to do with an independent risk factor of poor clinical outcomes [6–8] and is complicated by target organ damage, which various markers including left ventricular hypertrophy (LVH), carotid intima-media thickness (CIMT), a decline of estimated glomerular filtration rate (eGFR), albuminuria [2, 9–11]. Therefore, reducing cardiovascular risk and improving prognosis is crucial for patients with CKD by controlling hypertension.

Blood pressure variability (BPV) is described by blood pressure (BP) spontaneous oscillation and includes different time-phase variability from very short-term BPV (beat-by-beat), short-term BPV (over 24 h), mid-term BPV(day-to-day), and long-term BPV(visit-to-visit) [12]. The 24-h BP level, circadian rhythm, and BPV are three elements of perfect 24-h BP control that improve blood pressure management [13]. Recent studies demonstrate that short-term BPV was a better acute predictor that causes an atherothrombotic CVD events measured by ambulatory blood pressure monitoring (ABPM), and is generally less studied than long-term BPV assessed by office BP [14, 15].

Some previous evidence has proven that short-term BPV could be independent from mean BP values, related to the subclinical target organ damage and increased risk for of clinical events in hypertensive populations [16, 17]. A large Spanish cohort illustrated that an increase in short-term SBP-variability corresponded with advancing CKD stages which may be engaged with the sharp increase of cardiovascular risk with worsening renal function in hypertensive populations [18]. Nonetheless, patients with hypertension may be biased and may not represent the true BPV because of 41.4% were on antihypertensive treatment. Still there are controversies that increased short-term BPV seems to apparently result in target organ damage in CKD populations, besides the influence of the increase of the average blood pressure level. Prospective study of CKD stages1 to 4 patients observed that SBP wSD was independently related to the risk of renal adverse outcome [19]. Nevertheless, they did not elucidate an association with cardiovascular events. The importance of diastolic BPV being a risk factor for mortality and cardiovascular events was not evaluated and it needs further study to evaluate the association with diastolic BPV. Moreover, wSD was calculated by un-individualized diurnal/ nocturnal time interval used to assess SBP-variability which result in not get a sense of accuracy on real data. Unlike the above-mentioned studies, Recent studies illustrated that short-term BPV is not independently associated with renal diseases progression [20–22]. As far as we know, present evidence mainly concentrated in patients with hypertension and dialysis, remains controversial in the field of the above associations are important in pre-dialysis CKD patients at high risk for cardiovascular events. At the same time, it failed to find a routine clinical application in terms of the underlying mechanisms, methodological approach and clinical significance of the different types of BPV. Therefore, we conducted this study with the aim of investigating the relationship between short-term BPV and target organ damage in a Chinese non-dialysis CKD population.

## Methods

### Participants

**Fig. 1:**
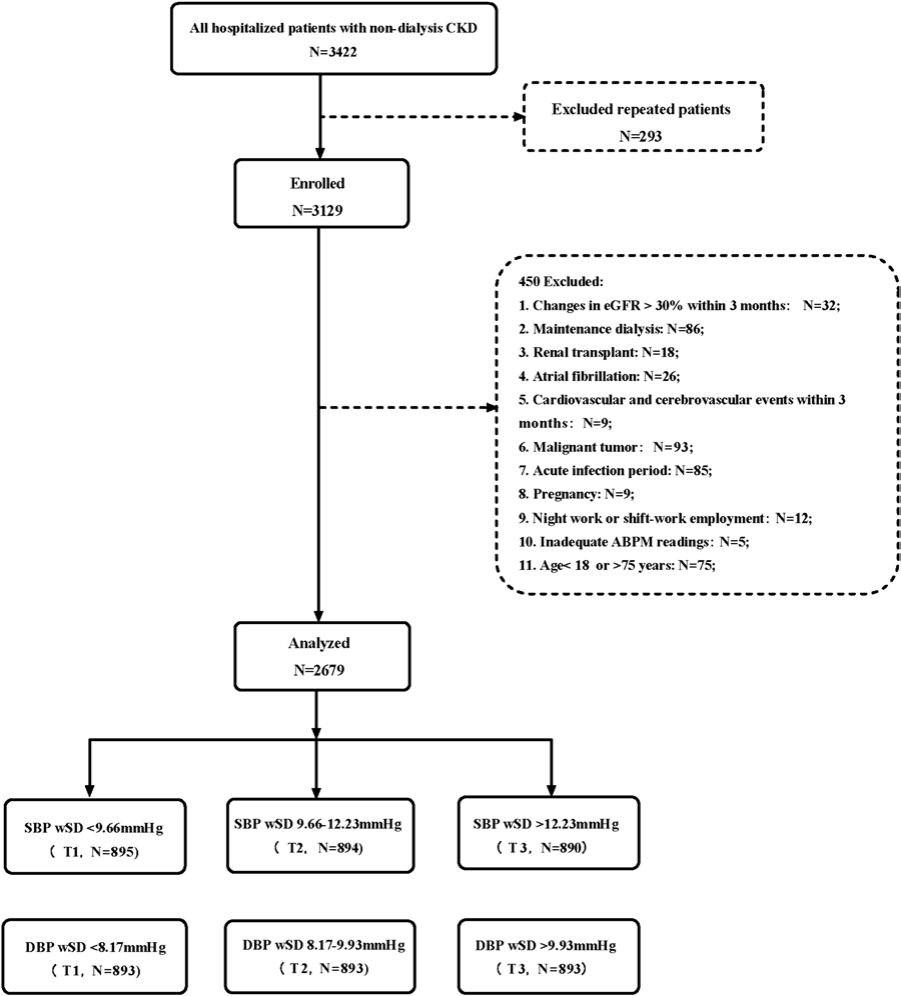
Flowchart of the participants. Flowchart depicting the selection of participating individuals for investigation. *ABPM* Ambulatory blood pressure monitoring, *DBP* Diastolic blood pressure, *SBP* Systolic blood pressure, *wSD* weighted standard deviation

The cross-sectional study enrolled all non-dialysis CKD including both men and women, aged 18–75 years, who were selected from the population of patients in the Department of Nephrology of the Fifth Affiliated Hospital of Sun Yat-sen University from November 2017 to July 2022. The study was open to 3129 patients who met all the inclusion criteria as specified in the *KDIGO guideline (2021)* [2]. In the course of their hospitalization, clinical data of patients are collected; Study protocols were reviewed and approved by institutional review boards or ethics committees, and all patients gave written informed consent.450 participants were excluded based on this criterion. Finally, total 2679 CKD patients were included in the analysis. The primary diseases were as follows: 1621 (60.5%) patients had chronic glomerulonephritis; 296 (11.0%) patients had diabetic kidney disease; 168 (6.3%) patients had hypertensive nephropathy; 84 (3.1%) patients had lupus nephropathy; and 510 (19.0%) patients had other causes. See Fig. 1 for the research flow chart selected by participants.

### Clinic blood pressure measurement

Clinic blood pressure (CBP) was measured by uniformly-trained nurses before wearing the wearable ABPM device. CBP was measured by averaging three measurements taken at 1–2 min intervals with an appropriately sized cuff, obtained from the uncovered right arm, after 5 min of sitting rest [9]. Hypertension by clinic was defined as clinic systolic blood pressure > 140 mmHg, and/or clinic diastolic blood pressure > 90 mmHg [9].

### Ambulatory blood pressure measurement

Participants were instructed that ABPM should be worn on and data collection was performed by nurses who had been trained. We defined daytime and nighttime intervals during ABPM based on self-reported awake and asleep times from the individual user's diary card [23]. Valid and reliable measurements were considered to be successful recorded for at least 70% of the expected 24-h readings [9, 23]. The degree of nocturnal BP dipping (%) was calculated as 100 × ($1-\frac{\mathrm{nighttime BP}}{\mathrm{daytime BP}}$). The following subgroups were classified: extreme dipper: > 20%; dipper: ≤ 20%, > 10%; non‐dipper: ≤ 10%, > 0%; riser: ≤ 0% [24, 25].

### Blood pressure variability measurement

We used wSD to quantify the short-term BPV, which was defined as the average SD of daytime and nighttime BP, and weight for the duration of the daytime and nighttime interval, respectively, with the following parameters: the standard deviation (SD) based on the formula: SD = $\sqrt{\frac{1}{N}{\sum }_{k=1}^{N}{({BP}_{k}-\overline{BP})}^{2}}$, the weighted standard deviation(wSD) was calculated as: wSD = $\frac{(daytimeSD\times daytime)+(nighttimeSD\times nighttime)}{24hperiod}$ [26]. All other indicators: coefficient of variation (CV), average real variability (ARV), average successive variation (ASV), time rate (TR) were also calculated together [27–29]. Where *N* is the number of valid BP readings and k is the number of the individual reading, $\overline{BP }$: the average BP during ABPM.

### Target organ damage assessment

#### Cardiac assessment

All image acquisitions and measurements were performed by experienced sonographers after receiving uniform train [30]. M-mode tracing was used to measure the interventricular septum, left ventricular internal diameter, and end-diastolic posterior wall thickness were measured from the parasternal long-axis window at the level of mitral valve leaflet tips to calculate left ventricular mas, with LVH was defined as > 115 g/m^2^ in men and > 95 g/m^2^ in women [9, 31].

#### Carotid ultrasonography

Measurement of the mean internal carotid intima-media wall thickness using B-mode ultrasonography (SonoSite, Bothell, WA). The measuring point was the area of the bilateral carotid arteries segment near the carotid bulb and free of plaques at end-diastole. All measurements were performed three times, and the average value was used [32]. As a sign of early atherosclerosis, CIMT was defined as carotid intima-media thickness > 0.9 mm [33].

#### Renal assessment

The CKD-EPI (Chronic Kidney Disease Epidemiology Collaboration) equation with serum creatinine was calibrated using an enzymatic, isotope dilution mass spectrometry traceable method used to estimate GFR [2]. A morning spot-urine sample was collected on the day to measure urinary albumin and creatinine by immunoturbidimetry for calculation of the urine albumin-to-creatinine ratio (UACR). For those with impaired renal function, including low eGFR (eGFR < 60 ml/ min/1.73 m^2^) and albuminuria (UACR > 300 mg/g) [34].

#### Covariate definition

Sociodemographic characteristics, medical history, lifestyle behaviors, and other clinical data were collected via interviews and medical records at the study entry. Body mass index (BMI) was calculated using the formula: BMI = weight (kg) / [height ^2^(m^2^)]. Smokers are defined as having smoked cigarettes for six months or more continuously or cumulatively in their lifetime and having smoked at least one cigarette in the 30 days preceding the survey. Alcohol users were defined as those who drank ≥ 1 time a week. Diabetes was defined by the self-reported history of a physician's diagnosis, use of anti-diabetes mellitus medication, or fasting plasma glucose > 126 mg/dL (200 mg/dL if nonfasting). History of CVD included pre-admission diagnosis of angina pectoris, myocardial infarction and stroke.

#### Statistical analysis

Normality of the data was tested using the Kolmogorov–Smirnov test. Continuous variables are expressed as mean ± SD for normally distributed variables and as median (interquartile range) for non–normally distributed variables. Baseline characteristics were presented as frequencies and proportions were computed to describe the distribution of categorical variables. One‐way ANOVA or nonparametric tests were used to compare data for continuous variables, which depends on the distribution of the data, while the chi‐squared test was used to compare data for categorical variables. The Bonferroni post hoc tests were used for pairwise multiple comparisons. In order to select the best indicators of short-term BPV, receiver operating characteristic (ROC) analysis was performed and the area under the curve (AUC) on ROC analysis was evaluated. Multivariable logistic regression analyses were subsequently used to explore the relationship between wSD with the target organ damage and other influencing factors. We further performed subgroup analyses to explore potential sources of heterogeneity based on age, sex, antihypertensive drug (AHD), CVD and DM history. Multiple imputations were used to maximize statistical power and minimize possible bias when excluding missing data from the analyses. Among the covariates, the proportion of missing data ranged from 0 to 6.7%. We used multiple imputations, based on 5 replications and a chained equation approach method in the R MI procedure, to account for missing data.*P* values < 0.05 was considered statistically significant. All statistical analyses were performed using SPSS version 25 (IBM Corp.) and R Version 3.6.0. Graphs were plotted with GraphPad Prism 9 (GraphPad Software Inc).

## Results

### Baseline characteristics

Supplemental Fig. 1 presents wSD was chosen as the indicators of short-term BPV owing to it has the best diagnostic accuracy which was divided into three groups based on tertile distribution according to equal numbers: T1(< 9.66 mmHg), T2(9.66–12.23 mmHg), and T3(> 12.23 mmHg) of SBPV; T1(< 8.17 mmHg), T2(8.17–9.93 mmHg), and T3(> 9.93 mmHg) of DBPV. The overall mean age was 47.53 ± 14.06 years and 56% of participants were male. The most frequently AHDs were calcium channel blocker (CCB) followed by angiotensin receptor blockers (ARB). The baseline eGFR was 69 ml/min/1.73 m^2^. Patients in the T2 or T3 group were all significantly older and had a higher level of BMI and more smokers, with a higher prevalence of diabetes mellitus and hypertension history, had a higher uric acid, 24-h mean SBP, 24-h mean DBP, clinic SBP, clinic DBP and hypertension by clinic, and a higher rate of taking antihypertensive drugs, but a lower eGFR than in the T1 group (*P-*trend < 0.05). On the cause of primary kidney disease, 1621 patients suffered from chronic glomerulonephritis; 296 patients suffered from diabetic kidney disease; 168 patients suffered from hypertensive nephropathy; 84 patients suffered from lupus nephritis; and 510 patients had other causes of renal disease (Table 1) (Supplemental Table 1).

**Table 1:** Baseline characteristics and study assignment of patients by tertiles of SBP-wSD

	**Overall (*N* = 2679)**	**Tertile of SBP-wSD**			
		**T1 (*N* = 895)**	**T2 (*N* = 894)**	**T3 (*N* = 890)**	***P*-trend value**
**Demographic**					
Age, y	47.53 ± 14.06	42.55 ± 12.77	47.26 ± 13.97 *	52.81 ± 13.52*‡	< 0.001
Male, *N* (%)	1501(56.0)	492(55.0)	515(57.6)	494(55.5)	0.495
BMI, kg/m^2^	24.18 ± 4.20	23.49 ± 4.25	24.22 ± 4.16 *	24.82 ± 4.08*‡	< 0.001
Current smoker, *N* (%)	505(18.9)	132(14.7)	182(20.4) *	191(21.5) *	0.001
Alcohol intake, *N* (%)	417(15.6)	136(15.2)	147(16.4)	134(15.1)	0.673
Diabetes mellitus, *N* (%)	592(22.1)	95(10.6)	176(19.7) *	321(36.1) *‡	< 0.001
CVD history, *N* (%)	384(14.3)	81(9.1)	114(12.8) *	189(21.2) *‡	< 0.001
Hypertension history, *N* (%)	1360(50.8)	303(33.9)	461(51.6) *	596(67.0)*‡	< 0.001
Statins, *N* (%)	711(26.5)	198(22.1)	223(24.9)	290(32.6)*‡	< 0.001
**Etiology of CKD**					
Glomerulonephritis, *N* (%)	1621(60.5)	618(69.1)	558(62.4) *	445(50.0) *‡	< 0.001
Diabetic kidney disease, *N* (%)	296(11.0)	33(3.7)	81(9.1) *	182(20.4) *‡	< 0.001
Hypertensive nephropathy, *N* (%)	168(6.3)	38(4.2)	52(5.8)	78(8.8) *‡	< 0.001
Lupus nephropathy, *N* (%)	84(3.1)	27(3.0)	24(2.7)	33(3.7)	0.449
Others, *N* (%)	510(19.0)	179(20.0)	179(20.0)	152(17.1)	0.190
**Laboratory**					
FBG, mmol/L	4.80(4.32,5.50)	4.64(4.24,5.14)	4.80(4.34,5.45)	5.00(4.45,6.00)*‡	< 0.001
Hemoglobin, g/L	124.66 ± 25.92	128.55 ± 23.58	126.39 ± 26.57	119.00 ± 26.55*‡	< 0.001
Serum albumin, g/L	38.63(33.40,42.00)	39.20(34.20,42.50)	39.00(33.75,42.35)	37.70(32.58,41.40)	0.007
Uric acid, mmol/L	425.64 ± 120.82	409.18 ± 119.08	429.48 ± 122.38 *	438.35 ± 119.26*	< 0.001
Cholesterol, mmol/L	4.74(3.93,5.89)	4.67(3.90,5.69)	4.78(3.94,5.87)	4.80(3.93,6.09)	0.935
HDL-C, mmol/L	1.10(0.90,1.37)	1.13(0.94,1.43)	1.10(0.89,1.38)	1.07(0.88,1.32)*	0.008
LDL-C, mmol/L	2.76(2.11,3.57)	2.73(2.14,3.48)	2.77(2.10,3.52)	2.77(2.11,3.77)	0.954
Serum calcium, mmol/L	2.15(2.02,2.25)	2.16(2.03,2.25)	2.15(2.02,2.25)	2.13(2.02,2.25)	0.643
Serum phosphate, mmol/L	1.09(0.94,1.28)	1.06(0.91,1.25)	1.09(0.94,1.28)	1.10 (0.95,1.33)	< 0.001
iPTH, pmol/L	5.11(3.35,9.30)	4.66(3.20,7.27)	4.88(3.21,9.44) *	6.02(3.72,11.75)*‡	< 0.001
Blood urea nitrogen, mmol/L	6.20(4.58,10.00)	5.50(4.20,8.10)	6.10(4.60,9.90) *	7.40(5.10,12.40)*‡	< 0.001
Serum creatinine, µmol/L	100.00(72.00,175.00)	89.00(67.00,137.00)	100.25 (72.00,176.25)*	116.00(80.75,226.00)*‡	< 0.001
eGFR, ml/min/1.73 m^2^	69.00(35.00,101.00)	85.00(51.00,108.00)	70.00 (34.00,102.00)*	55.00(24.00,88.00)*‡	< 0.001
UACR, mg/g	475.88(60.19,1744.94)	308.91(34.74,1410.84)	407.53(58.60,1528.00)	756.31(93.23,2456.83)*‡	< 0.001
**Blood pressure indices**					
Clinic-SBP, mmHg	135.43 ± 23.87	124.91 ± 19.21	135.07 ± 22.57*	146.38 ± 24.54*‡	< 0.001
Clinic-DBP, mmHg	85.62 ± 14.46	82.24 ± 12.71	86.31 ± 13.98*	88.33 ± 15.88*‡	< 0.001
24 h-mean SBP, mmHg	124.10 ± 16.21	115.59 ± 12.27	122.38 ± 14.03*	134.37 ± 16.15*‡	< 0.001
24 h-mean DBP, mmHg	81.09 ± 11.72	77.47 ± 10.35	80.66 ± 11.21*	85.17 ± 12.23*‡	< 0.001
Hypertension by clinic-SBP, *N* (%)	1052(39.3)	176(19.7)	343(38.4)*	533(59.9)*‡	< 0.001
Hypertension by clinic-DBP, *N* (%)	950(35.5)	230(25.7)	331(37.0)*	389(43.7)*‡	< 0.001
24 h-SBP SD, mmHg	12.41 ± 3.70	9.08 ± 1.39	11.81 ± 1.27*	16.36 ± 3.25*‡	< 0.001
24 h-DBP SD, mmHg	10.08 ± 3.26	8.41 ± 1.64	9.91 ± 1.79*	11.94 ± 4.46*‡	< 0.001
Daytime-SBP SD, mmHg	12.20 ± 3.87	8.71 ± 1.34	11.56 ± 1.28*	16.35 ± 3.42*‡	< 0.001
Daytime-DBP SD, mmHg	9.82 ± 3.00	8.10 ± 1.70	9.73 ± 3.22*	11.65 ± 2.72*‡	< 0.001
Nighttime-SBP SD, mmHg	9.41 ± 3.54	7.14 ± 2.13	9.15 ± 2.34*	11.94 ± 4.00*‡	< 0.001
Nighttime-DBP SD, mmHg	7.90 ± 2.83	6.53 ± 2.16	7.89 ± 2.37*	9.29 ± 3.14*‡	< 0.001
SBP wSD, mmHg	11.41 ± 3.32	8.28 ± 1.02	10.88 ± 0.74*	15.10 ± 2.78*‡	< 0.001
DBP wSD, mmHg	9.28 ± 2.46	7.67 ± 1.44	9.19 ± 2.33*	10.99 ± 2.26*‡	< 0.001
Dippers, *N* (%)	466(17.4)	118(13.2)	176(19.7)*	172(19.3)*	< 0.001
Non-dippers, *N* (%)	1562(58.3)	611(68.3)	519(58.1)*	432(48.5)*‡	< 0.001
Risers, *N* (%)	645(24.1)	169(18.9)	199(22.3)	277(31.1)*‡	< 0.001
Extreme dippers, *N* (%)	17(0.6)	1(0.1)	4(0.4)	12(1.3)*	0.003
**Antihypertensive treatment**					
Antihypertension drugs, *N* (%)	1108(41.4)	252(28.2)	365(40.8) *	491(55.2) *‡	< 0.001
ACEI, *N* (%)	142(5.3)	51(5.7)	48(5.4)	43(4.8)	0.712
ARB, *N* (%)	753(28.1)	244(27.3)	264(29.5)	245(27.5)	0.507
β-blockers, *N* (%)	477(17.8)	105(11.7)	160(17.9)*	212(23.8)*‡	< 0.001
Calcium channel blockers, *N* (%)	1153(43.0)	244(27.3)	390(43.6)*	519(58.3)*‡	< 0.001
Others, *N* (%)	193(7.2)	29(3.2)	52(5.8)*	112(12.6)*‡	< 0.001

Data are presented as numbers and percentages, means and standard deviations, or median and quartile ranges. *ACEI* Angiotensin-converting enzyme inhibitor, *ARB* Angiotensin receptor blocker, *BMI* Body mass index, *CVD* Cardiovascular disease, *DBP* Diastolic blood pressure, *GFR* Glomerular filtration rate, *FBG* Blood fasting glucose, *HDL-C* High-density lipoprotein cholesterol, *iPTH*, Intact parathyroid hormone, *LDL-C* Low-density lipoprotein cholesterol, *wSD* Weighted standard deviation, *SBP* Systolic blood pressure, *SD* Standard deviation, *UACR* Urinary albumin-to-creatinine ratio

^*^p < 0.05 compared with T1

^‡^*p* < 0.05 compared withT2

### Prevalence of target organ damage

**Fig. 2:**
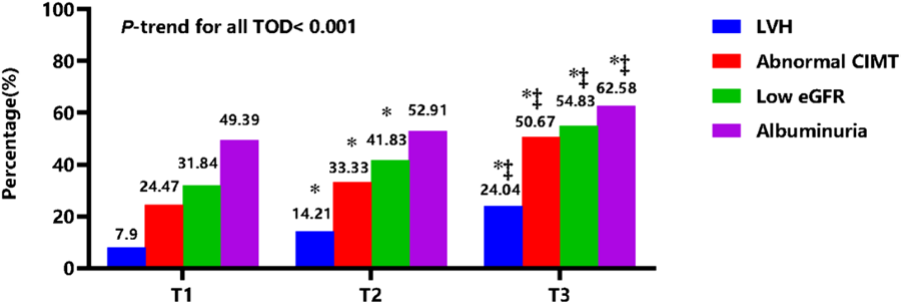
Comparison of target organ damages in tertiles of SBP wSD groups. *LVH* Left ventricular hypertrophy, *CIMT* Carotid intima-media thickness, *GFR* Glomerular filtration rate, *TOD* Target organ damage *P < 0.05 compared with T1 ‡ *P* < 0.05 compared with T2

The overall prevalence of LVH, abnormal CIMT, low eGFR, and albuminuria in patients with SBP wSD was 15.38%, 36.13%, 42.81%, and 54.95%, respectively. Patients with T3 group had a higher prevalence of LVH (24.04%), abnormal CIMT (50.67%), low eGFR (54.83%), and albuminuria (62.58%) compared with those in the T1 or T2 groups. The difference in the proportion of target organ damage between the three groups showed a linear trend, similar to DBP wSD (*P-*trend < 0.05) (Fig. 2) (Supplemental Fig. 2).

### BPV in different CKD stages

**Fig. 3:**
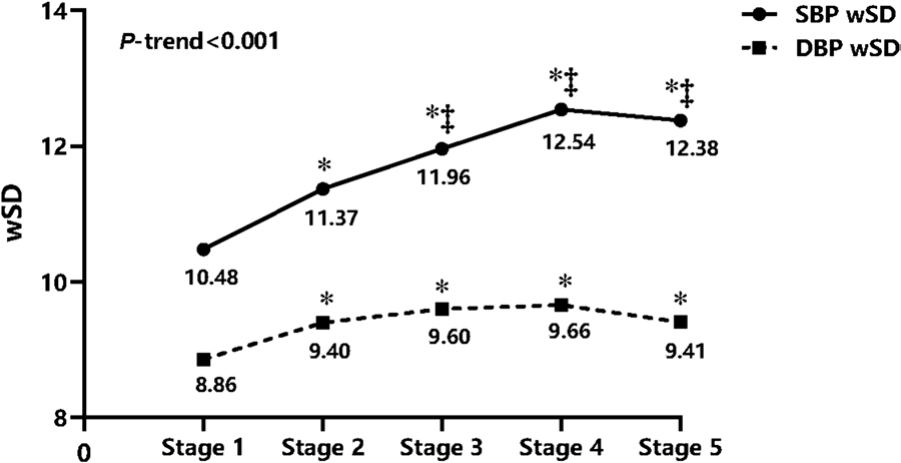
BPV in different CKD stages. Blood pressure variability parameters for 24-h ambulatory SBP and DBP according to CKD Stages. *DBP* Diastolic blood pressure, *SBP* Systolic blood pressure, *wSD* Weighted standard deviation *P < 0.05 compared with Stage1 ‡*P* < 0.05 compared with Stage2

Across CKD stages, a progressively increased level of wSD with the advancement of CKD except in stage5. The SBP wSD of CKD from stage1to5 was 10.48 ± 2.79 mmHg, 11.37 ± 3.20 mmHg,11.96 ± 3.48 mmHg,12.54 ± 3.73 mmHg,12.38 ± 3.54 mmHg, and similar DBP wSD was 8.86 ± 2.70 mmHg,9.40 ± 2.20 mmHg,9.60 ± 2.23 mmHg, 9.66 ± 2.45 mmHg, 9.41 ± 2.38 mmHg. There was a linear trend between the level of wSD and the different CKD stages (*P-*trend < 0.001) (Fig. 3).

### Factors associated with BPV in patients with CKD

Supplemental Table 2 presents the multivariate regression analyses, including T3(> 12.23 mmHg) was taken as dependent variable, and several demographics, clinical and laboratory factors that can interfere with BPV were taken as independent variables. Of the parameters tested, older age, male gender, higher level of BMI, more smokers, diabetes and use of AHD were independently related to the elevated SBP wSD adjusting for the other confounding factors (*P* < 0.05). In the end, the increase of clinic SBP was related to the higher OR of increased SBP wSD, similar to what was observed in the total population increasing clinic DBP with higher OR of increased DBP wSD (*P* < 0.001).

**Table 2:** Trends in the association between tertiles of wSD groups and TOD

		**LVH**		**Abnormal CIMT**		**Low eGFR**		**Albuminuria**	
		**OR (95%CI)**	***P* value**	**OR (95%CI)**	***P* value**	**OR (95%CI)**	***P* value**	**OR (95%CI)**	***P* value**
**SBP_wSD**									
T1 (8.51[< 9.66 mmHg])	895	Ref		Ref		Ref		Ref	
T2 (10.87[9.66—12.23 mmHg])	894	0.93(0.71, 1.22)	0.604	0.84(0.68, 1.04)	0.108	0.96(0.80, 1.15)	0.635	0.92(0.75, 1.13)	0.441
T3 (14.25[> 12.23 mmHg])	890	1.29(0.99, 1.68)	0.059	1.34(1.07, 1.67)	0.011	1.21(0.99, 1.47)	0.062	1.31(1.05, 1.63)	0.019
**DBP_wSD**									
T1 (7.20[< 8.17 mmHg])	893	Ref		Ref		Ref		Ref	
T2 (9.03[8.17 – 9.93 mmHg])	893	0.99(0.77, 1.29)	0.966	0.84(0.68, 1.04)	0.100	0.87(0.73, 1.05)	0.141	0.84(0.68, 1.02)	0.084
T3 (11.28[> 9.93 mmHg])	893	1.24(0.96, 1.60)	0.103	1.39(1.13, 1.72)	0.002	1.25(1.04, 1.51)	0.019	1.33(1.08, 1.64)	0.008

*CIMT* carotid intima-media thickness, *DBP* diastolic blood pressure, *GFR* Glomerular filtration rate, *LVH* Left ventricular hypertrophy, *wSD* Weighted standard deviation, *SBP* Systolic blood pressure

### Association between BPV and target organ damage

**Fig. 4:**
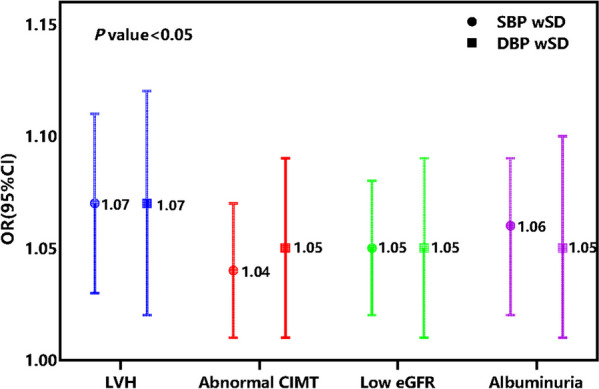
Multivariable logistic regression analysis for BPV types and target organ damage. The OR of BPV for LVH was adjusted for age, gender, BMI, cardiovascular disease history, antihypertensive drugs, serum albumin, eGFR, hemoglobin, FBG, serum albumin, SBP wSD /DBP wSD, and hypertension by clinic-SBP/hypertension by clinic-DBP. The OR of BPV for abnormal CIMT was adjusted for age, gender, current smoking, alcohol intake, cardiovascular disease history, FBG, LDL-C, SBP wSD /DBP wSD and hypertension by clinic-SBP/hypertension by clinic-DBP. The OR of BPV for low eGFR was adjusted for age, gender, BMI, current smoking, alcohol intake, diabetes mellitus, use of antihypertension drugs, cholesterol, LDL-C, serum albumin, SBP wSD /DBP wSD, and hypertension by clinic-SBP/hypertension by clinic-DBP. The OR of BPV for albuminuria was adjusted for age, gender, diabetes mellitus, HDL-C, serum albumin, iPTH, blood urea nitrogen, serum calcium, SBP wSD /DBP wSD, and hypertension by clinic-SBP/hypertension by clinic-DBP. *BMI* Body mass index, *CIMT* Carotid intima-media thickness, *DBP* Diastolic blood pressure, *GFR* Glomerular filtration rate, FBG Blood fasting glucose, *HDL-C* High-density lipoprotein cholesterol, *iPTH* Intact parathyroid hormone, *LDL-C* Low-density lipoprotein cholesterol, *LVH* Left ventricular hypertrophy, *wSD* Weighted standard deviation, *SBP* systolic blood pressure

Multivariate logistic regression analyses demonstrated that wSD was related to subclinical target organ damage whilst additionally adjusting for the other confounding factors independent of the average BP value level in categorical form. The OR of the SBP wSD was (1.07 [1.03,1.11], *P* < 0.001) for LVH, (1.04 [1.01,1.07, *P* = 0.029) for abnormal CIMT, (1.05 [1.02,1.08], *P* = 0.002) for low eGFR, and (1.06 [1.02,1.09], *P* = 0.002) for albuminuria; The OR of the DBP wSD was (1.07 [1.02,1.12], *P* = 0.005) for LVH, (1.05 [1.01,1.09], *P* = 0.028) for abnormal CIMT, (1.05 [1.01,1.09], *P* = 0.022) for low eGFR, and (1.05 [1.01,1.10], *P* = 0.025) for albuminuria when adjusted for confounding factors and mean BP (Fig. 4) (Supplemental Table 3). The trend test result indicates that the association between abnormal CIMT or albuminuria and wSD could increase closely with increment BPV levels compared to T1 group (Table 2). All *P*-values < 0.05 were considered significant.

### Subgroup analysis

Subgroup analysis was examined according to demographic variables and potential confounders. Results showed that the relationship of wSD with target organ damage remained different from that found in the various groups in multivariate analysis (Supplemental Fig. 3). All *P*-values < 0.05 were considered significant.

## Discussion

In this cross-sectional study, we investigated associations between wSD with subclinical target organ damage in a large sample of patients with Chinese non-dialysis CKD. Meanwhile, BMI, smoker, AHD use, 24-h CBP were significantly associated with wSD. We observed a risk tendency towards higher prevalence of target organ damage increased as BPV upgraded. The participants with the higher wSD with the advancement of renal injury. Short-term BPV was related with the increased risk of LVH, abnormal CIMT, low eGFR, and albuminuria independent of average BP levels when adjusted for confounding factors and mean BP. Subgroup analysis indicated that wSD was affected by anti-hypertension medication in multivariate analyses, except for the correlation with LVH of short-term diastolic BPV. Consequently, this research suggests a potential effect for short-term BPV is substantially associated with for adverse events in patients with non-dialysis CKD.

Several pioneering investigations have shown that in hypertension populations, short-term BPV is related to an increased risk of organ damage and poor prognosis in a manner that is irresponsible of average BP values [17, 35, 36]. Triantafyllidi and colleagues have clearly shown that the short-term BPV reduction predicted LVMI improvement independently of BP levels [37]. However, It is not yet clear whether this elevated variability is a contributor to the development and progression of the disease, a byproduct of the disease, or wholly epiphenomenal to it. There is currently little knowledge about the relationships between longitudinally evaluated differences in short-term BPV and the changes in organ damage or adverse clinical events in CKD populations, and these findings, are not consistent. A cohort study from China, 693 were excluded owing to miss data regarding BPV values among 2114 enrolled patients which could cause selection bias. The study also assessed BPV from one measurement and showed association only with renal adverse outcome and not find an association with cardiovascular events [19]. Although it has been shown previously that short-term BPV was regarded as an acute risk factor which leads to cardiovascular events based on chronically advancing LVH because of an abrupt increase in SBP [15]. However, an Italian study showed that 24-h ambulatory BPV do not accurately predict the risk of progression of CKD [20]. Ryu and colleagues also reported that SBP ARV was not associated kidney injury in a large sample of in Korean hypertensive CKD patients [22]. Possibly, when assessing different populations, races, and assessment methods, the differences between study results may show differences in the risk of BPV. As part of our current research, we have explored relationship between short-term BPV with subclinical target organ damage of CKD patients in large sample size, which have consequences of the association not depend on absolute BP values but on the level of short-term BPV. Our study had sufficient data directly comparing short-term BPV of all these indicators. It may be indicated that wSD should be preferred, considering that wSD had the highest AUC value compare other indicators, meanwhile, can not only reflects the levels of nighttime blood pressure variation but also reduces the confounding effects of day-night BP fluctuations. Comparison of degree of target organ damage and various indicators of patients there are a few studies showing results that intima media thickness have the closest relationship with ARV or TR, is the better early predictor for arteriosclerosis [38, 39]. The present study indicated that wSD closely related to CIMT, macroalbuminuria, low eGFR in DBP wSD, not low eGFR in SBP wSD and LVH. Such a discrepant result may be related to the heterogeneity of the ABPM methodology used in the different countries that pooled the ABPM data. Longitudinal studies are warranted to elucidate the issues regarding the association and whether antihypertensive treatment strategies should be primarily targeted at the degree of BPV.

In current study, we analyzed clinical factors that BMI, smoker, AHD use, and CBP were associated with wSD, at the same time, findings in study showing that nondippers accounts for a considerable proportion of circadian rhythm, and the proportionality of risers elevated with higher wSD. Those findings in line with previous study proved that are nondippers and risers associated with organ damage and poor CV prognoses in CKD patients [40–42]. However, nondippers slightly decreases with a trend of wSD elevation were opposite of what we expected. We regard it as likely, however, there are some reasons that may explain this observation. The study is limited first by antihypertensive treatment interfere accompanied by elevated short-term BPV. Secondly, this matched the previously reported findings, a non-dipping blood pressure pattern does not predict the risk of renal outcomes in CKD patients [43].

A few mechanisms have been proposed to illustrate the increased short-term BPV and not only may suggest a potential pathophysiologic, but also predict progression of subclinical diseases that have a direct effect on organ damage, including increased central sympathetic drive, reduced arterial and cardiopulmonary reflex, humoral and rheological factors, behavioral and emotional factors, activity and sleep, reduced arterial compliance, increased genetic variation, and improper dosing or titration of AHD et al., however, the specific mechanism is not yet clear [44–50]. Kazuomi Kario and colleagues recently proposed that short-term BPV has been considered as an acute risk factor for mechanical stress-induced plaque rupture leading to cardiovascular events. Kario et al. [13] and Kario [15], which urgently needed to provide a new hemodynamic biomarker-initiated way to prevent CVD events in combination with the traditional prevention of chronic risk factor and needs to be further validated in large prospective clinical trials to clarify the role of BPV in the development of CKD and the mechanisms involved. The existing evidences proved that additional cardiovascular protection can be obtained by reducing BPV. Therefore, in order to optimize cardiovascular protection, if it is a cause, then treatment with CCB, not only reducing BP but also lowering BPV, should be superior to other treatments [13].

### Study limitations

Strengths of this study include the relatively large sample size and the first extensive evaluation of subclinical cardiovascular and renal injury in the association of short-term BPV in Chinese non-dialysis CKD patients in this cross-sectional study. Nevertheless, some limitations of the current study need to be considered. First, this result may be influenced by the cross-sectional nature of this study, which limits the assessment of clarifying the cause-effect relationship. Second, our analyses were restricted to ethnically Chinese patients, and it is unclear whether our findings could be extrapolated to other racial/ethnic groups. Third, in fact, antihypertensive medication is a confounding factor in BP measurements and 41.4% of patients were taking antihypertensive medication before enrollment, and we were unable to assess the association between target organ damage and/or BPV and the use of AHD. Although we carefully adjusted BP for the treatment effect of antihypertensives in multivariate analyses and performed a subgroup analysis to assess those patients without AHDs. Fourth, the short-term BPV was assessed due to the data acquired by ABPM in this study, and the long-term BPV has not been measured in patients, hence, cannot compare the short-term BPV and long-term BPV. Thus, additional research is required to define or specify essential elements of clinical diagnostic assessment. Further research is needed to determine the optimal threshold for this analysis and identify a number of clear targets for therapeutic interventions.

## Conclusions

In conclusion, the results of our study showed that short-term BPV and, more specifically, wSD was significantly and positively associated with the risk of subclinical target organ damage in Chinese non-dialysis CKD populations in this cross-sectional study, even after adjustment for BP levels and vascular risk factors. We strongly emphasize the warranty for further studies to clarify underlying mechanism and causality associations between them, and a well-controlled short-term BPV should be prioritized in the management of hypertension combined with the data on organ damage and psycho-behavioral, genomic, environmental, and nutritional risk factors, has the potential to achieve a perfect individualized medicine regimen to reduce the occurrence of major CVD events.

### Supplementary Information

**Supplementary Material 1.**

## Data Availability

Data is available on request sent to the corresponding author.

## Abbreviations

CKD: Chronic Kidney Disease
CVD: Cardiovascular Disease
LVH: Left Ventricular Hypertrophy
CIMT: Carotid Intima-media Thickness
GFR: Glomerular Filtration Rate
BP: Blood Pressure
BPV: Blood Pressure Variability
SD: Standard Deviation
wSD: Weighted Standard Deviation
CV: Coefficient of Variation
ARV: Average Real Variability
ASV: Average Successive Variation
TR: Time Rate
UACR: Urine Albumin-to-creatinine Ratio
BMI: Body Mass Index
ROC: Receiver Operating Characteristic
AUC: Area Under the Curve
AHD: Antihypertensive Drug
CCB: Calcium Channel Blocker
ARB: Angiotensin Receptor Blockers
iPTH: Intact Parathyroid Hormone
