# Supplementary material for: Association between short-term blood pressure variability and target organ damage in non-dialysis patients with chronic kidney disease
Source: BMC Nephrol. 2024 Mar 21;25:111. doi: 10.1186/s12882-024-03541-x (PMC10958852; doi:10.1186/s12882-024-03541-x)
Supplement: Supplementary file 1 — Supplementary Material 1. [file 12882_2024_3541_MOESM1_ESM.pdf]

Supplemental Fig.1 Different short-term BPV indicators of ROC curves in various target organ damage

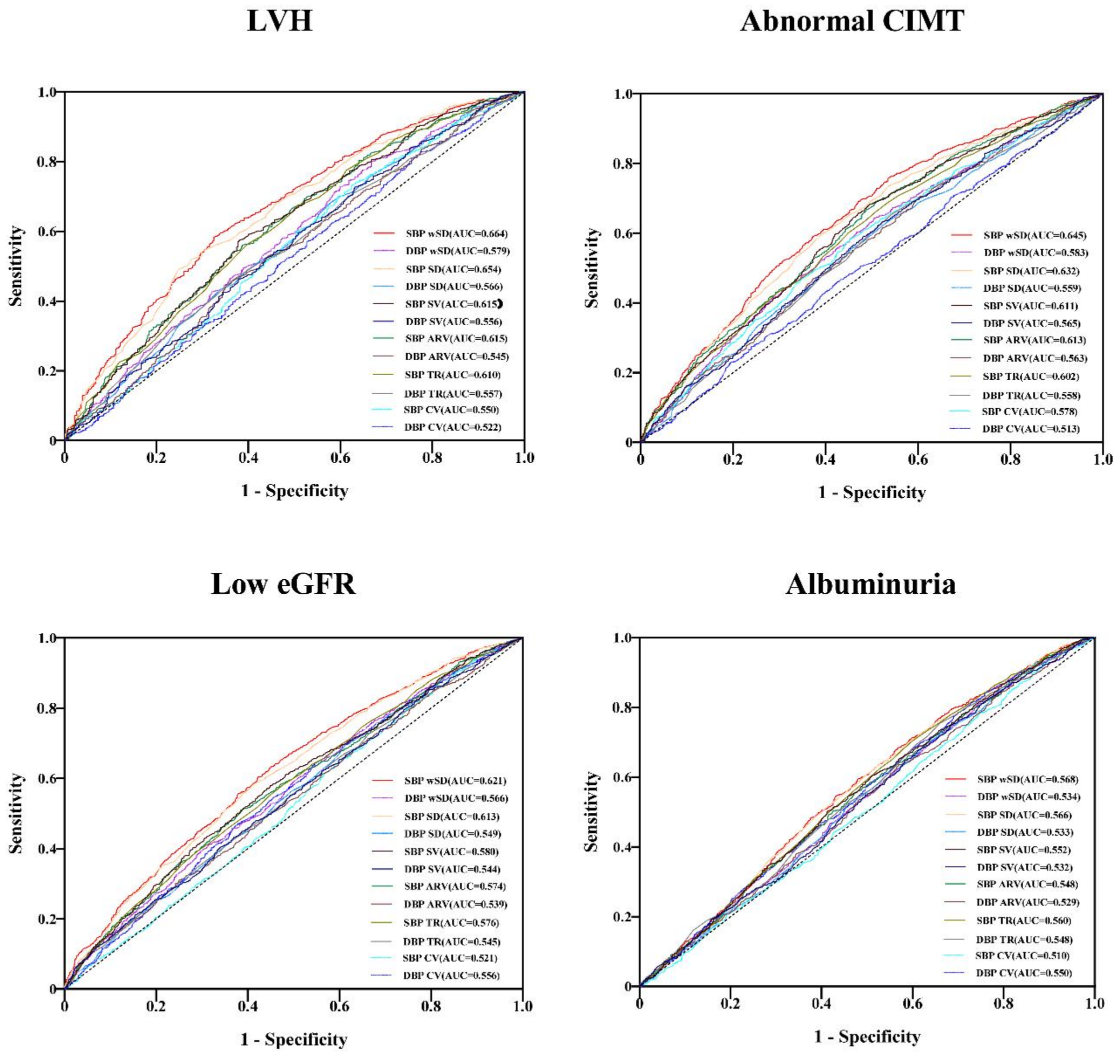

ARV Average real variability, *ASV* Average successive variation, *CIMT* Carotid intima-media thickness, *CV* Coefficient of variation, *DBP* Diastolic blood pressure, *GFR* Glomerular filtration rate, *LVH* Left ventricular hypertrophy, *SBP* Systolic blood pressure, *SD* Standard deviation, *TR* Time rate; *wSD* Weighted standard deviation

Supplemental Table 1 Baseline characteristics and study assignment of patients by tertiles of DBP-wSD

	Overall（N =2679）	Tertiles of DBP-wSD			*P*-trend value
		T1（N =893）	T2（N =893）	T3（N =893）	
**Demographic**					
Age, y	47.53±14.06	44.91±14.43	48.83±13.99 *	48.85±13.40 *	<0.001
Male, *N* (%)	1501（56.0）	444（49.7）	518（58.0）*	539（60.4）*	<0.001
BMI, kg/m^2	24.18±4.20	23.03±3.90	24.22±4.06 *	25.28±4.31 *‡	<0.001
Current smoker, *N* (%)	505（18.9）	106（11.9）	183（20.5）*	216（24.2）*	<0.001
Alcohol intake, *N* (%)	417（15.6）	116（13.0）	140（15.7）	161（18.0）*	0.013
Diabetes mellitus, *N* (%)	592（22.1）	146（16.3）	203（22.7）*	243（27.2）*	<0.001
CVD history, *N* (%)	384（14.3）	107（12.0）	113（12.7）	164（18.4）*‡	<0.001
Hypertension history, *N* (%)	1360(50.8)	335 (37.5)	452(50.7) *	573(64.2) *‡	<0.001
Statins, *N* (%)	711(26.5)	205 (23.0)	219(24.5)	287(32.1) *‡	<0.001
**Etiology of CKD**					
Glomerulonephritis, *N* (%)	1621（60.5）	585（65.5）	524（58.7）*	512（57.3）*	0.001
Diabetic nephropathy, *N* (%)	296（11.0）	79（8.8）	101（11.3）	116（13.0）*	0.019
Hypertensive nephropathy, *N* (%)	168（6.3）	42（4.7）	55（6.2）	71（8.0）*	0.018
Lupus nephropathy, *N* (%)	84（3.1）	33（3.7）	27（3.0）	24（2.7）	0.461
Others, *N* (%)	510（19.0）	154（17.2）	186（20.8）	170（19.0）	0.156
**Laboratory**					
FBG, mmol/L	4.80(4.32,5.50)	4.74(4.30,5.39)	4.80(4.35,5.50)	4.90(4.38,5.70)	0.104
Hemoglobin, g/L	124.66±25.92	123.67±25.59	124.79±25.09	125.51±27.02	0.325
Serum albumin, g/L	38.63(33.40,42.00)	38.10(32.85,41.80)	38.80(33.40,42.20)	39.11(33.70,42.30)	0.137
Uric acid, mmol/L	425.64±120.82	406.46±121.37	425.13±114.25 *	445.31±123.65 *‡	<0.001
Cholesterol, mmol/L	4.74(3.93,5.89)	4.72 (3.94,5.88)	4.69(3.85,5.60)	4.84(3.99,6.14)	0.045
HDL-C, mmol/L	1.10(0.90,1.37)	1.15(0.94,1.45)	1.08(0.89,1.36) *	1.07(0.88,1.31) *	0.010
LDL-C, mmol/L	2.76(2.11,3.57)	2.72(2.10,3.52)	2.73(2.11,3.48)	2.80(2.15,3.81)	0.625
Serum calcium, mmol/L	2.15(2.02,2.25)	2.14(2.01,2.25)	2.15(2.01,2.25)	2.15(2.04,2.25)	0.318
Serum phosphate, mmol/L	1.09(0.94,1.28)	1.09(0.94,1.28)	1.07(0.92,1.27)	1.10 (0.94,1.30)	0.165
iPTH, pmol/L	5.11(3.35,9.30)	4.84(3.21,8.55)	4.88(3.24,8.77)	5.79(3.68,10.23) *	0.026
Blood urea nitrogen, mmol/L	6.20(4.58,10.00)	5.70(4.29,9.45)	6.10(4.60,9.60)	6.80(4.90,10.93) *‡	<0.001
Serum creatinine, μmol/L	100.00(72.00,175.00)	91.00(66.50,159.00)	100.00 (71.60,167.25)	109.00(79.00,202.00) *‡	<0.001
eGFR, ml/min/1.73 m2	69.00(35.00,101.00)	80.50(41.00,107.00)	69.50 (38.00,100.00) *	60.00(29.00,94.00) *‡	<0.001
UACR, mg/g	475.88(60.19,1744.94)	403.85(70.68,1740.73)	392.72(44.71,1498.17)	605.43(76.28,2061.16) ‡	0.006
**Blood pressure indices**					
Clinic-SBP, mmHg	135.43±23.87	128.16±21.87	135.35±23.13 *	142.79±24.30 *‡	<0.001
Clinic-DBP, mmHg	85.62±14.46	81.52±12.75	85.20±13.41 *	90.15±15.76 *‡	<0.001
24 hour-mean SBP, mmHg	124.10±16.21	117.33±14.04	123.60±15.29 *	131.35±16.10 *‡	<0.001
24 hour-mean DBP, mmHg	81.09±11.72	76.66±10.67	80.64±10.82 *	85.97±11.74 *‡	<0.001
Hypertension by clinic-SBP, *N* (%)	1052(39.3)	232(26.0)	359(40.2) *	461(51.6) *‡	<0.001
Hypertension by clinic-DBP, *N* (%)	950(35.5)	206 (23.1)	317(35.5) *	427 (47.8) *‡	<0.001
24 hour-SBP SD, mmHg	12.41±3.70	10.02±2.42	12.08±2.73 *	15.13±3.82 *‡	<0.001
24 hour-DBP SD, mmHg	10.08±3.26	7.73±1.19	9.79±0.96*	12.73±4.13 *‡	<0.001
Daytime-SBP SD, mmHg	12.20±3.87	9.79±2.58	11.84±2.91 *	14.96±4.02 *‡	<0.001

Daytime-DBP SD, mmHg	9.82±3.00	7.40±1.16	9.50±1.00 *	12.56±3.34 *‡	<0.001
Nighttime-SBP SD, mmHg	9.41±3.54	7.74±2.69	9.33±2.79 *	11.15±4.11 *‡	<0.001
Nighttime-DBP SD, mmHg	7.90±2.83	6.03±1.84	7.84±2.07 *	9.84±3.00 *‡	<0.001
SBP wSD, mmHg	11.41±3.32	9.21±2.18	11.13±2.37 *	13.90±3.40 *‡	<0.001
DBP wSD, mmHg	9.28±2.46	7.01±0.89	9.03±0.50 *	11.80±2.36 *‡	<0.001
Dippers, *N* (%)	727(27.1)	227(25.4)	249(27.9)	251(28.1)	0.366
Non-dippers, *N* (%)	1358(50.7)	480(53.8)	481(53.9)	397(44.5) *‡	<0.001
Risers, *N* (%)	518(19.3)	176(19.7)	143(16.0)	199(22.3) ‡	0.003
Extreme dippers, *N* (%)	93(3.5)	13(1.5)	29(3.2) *	51(5.7) *‡	<0.001
**Antihypertensive treatment**					
Antihypertension drugs, *N* (%)	1108（41.4）	279（31.2）	383（42.9）*	446（49.9）*‡	<0.001
ACEI, *N* (%)	142(5.3)	60(6.7)	38(4.3)	44(4.9)	0.056
ARB, *N* (%)	753(28.1)	227(25.4)	276(30.9) *	250(28.0)	0.036
β-blockers, *N* (%)	477(17.8)	122(13.7)	153 (17.1)	202(22.6) *‡	<0.001
Calcium channel blockers, *N* (%)	1153(43.0)	283(31.7)	378(42.3) *	492(55.1) *‡	<0.001
Others, *N* (%)	193(7.2)	35(3.9)	62(6.9) *	96(10.8) *‡	<0.001

Data are presented as numbers and percentages, means and standard deviations, or median and quartile ranges. *ACEI* Angiotensin-converting enzyme inhibitor, *ARB* Angiotensin receptor blocker, *BMI* Body mass index, *CVD* Cardiovascular disease, *DBP* Diastolic blood pressure, *GFR* Gstimated glomerular filtration rate, *FBG* Blood fasting glucose, HDL-C High-density lipoprotein cholesterol, *iPTH* Intact parathyroid hormone, *LDL-C* Low-density lipoprotein cholesterol, *wSD* Weighted standard deviation, *SBP* Systolic blood pressure, *SD* Standard deviation, *UACR* Urinary albumin-to-creatinine ratio

*$p < 0.05$ compared with T1

‡ $p < 0.05$ compared withT2

Supplemental Fig.2 Comparison of target organ damages in tertiles of DBP wSD groups

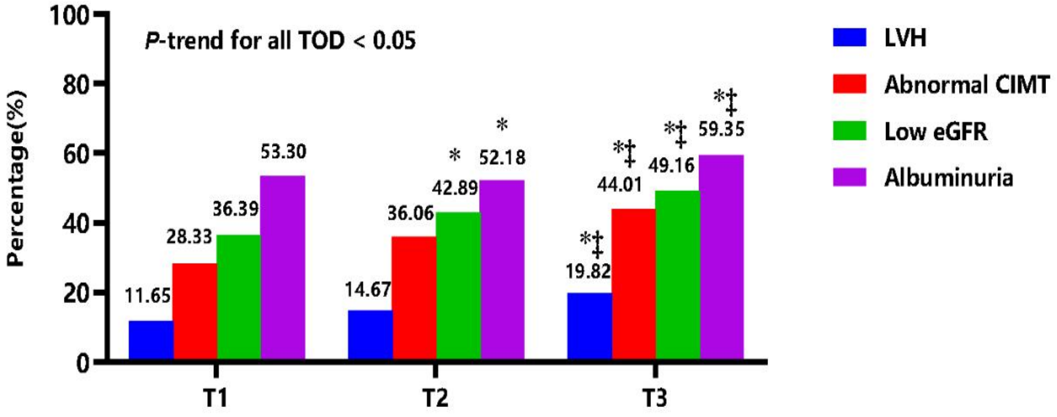

LVH Left ventricular hypertrophy, *CIMT* Carotid intima-media thickness, *GFR* Glomerular filtration rate, *TOD* Target organ damage

*$P < 0.05$ compared with T1

‡ $P < 0.05$ compared with T2

Supplemental Table 2 Multivariate regression analysis for wSD in patients with CKD

	SBP wSD		DBP wSD	
	OR（95%CI）	*P* value	OR（95%CI）	*P* value
Age, y	1.02（1.02,1.03）	<0.001	1.00（1.00,1.01）	0.419
Male gender, *N* (%)	0.71（0.58,0.86）	0.001	0.96（0.79,1.16）	0.676
BMI, kg/m^2	1.02（1.00,1.05）	0.045	1.08（1.05,1.10）	<0.001
Smoking, *N* (%)	1.29（1.01,1.64）	0.042	1.45（1.15,1.82）	0.002
Diabetes mellitus, *N* (%)	1.88（1.51,2.34）	<0.001	1.24（1.00,1.54）	0.052
CVD history, *N* (%)	1.20（0.94,1.55）	0.146	1.24（0.97,1.58）	0.087
Antihypertension drugs, N (%)	1.22（1.00,1.48）	0.047	1.23（1.02,1.48）	0.032
Cholesterol, mmol/L	0.94（0.85,1.03）	0.190	1.08（1.00,1.18）	0.062
LDL-C, mmol/L	1.12（1.00,1.26）	0.053	0.93（0.83,1.04）	0.189
Clinic SBP, mmHg	1.03（1.03,1.04）	<0.001	1.00（1.00,1.01）	0.152

Clinic DBP, mmHg	0.99 (0.98,1.00)	0.009	1.03 (1.02,1.04)	<0.001

BMI Body mass index, *CI* Confidence interval, *CVD* Cardiovascular disease, *LDL-C* Low-density lipoprotein cholesterol, *OR* Odds ratio, *DBP* Diastolic blood pressure, *wSD* Weighted standard deviation, *SBP* Systolic blood pressure

Supplemental Table 3 Multivariable logistic regression analysis for BPV types and target organ damage in the different models

	LVH		Abnormal CIMT		Low eGFR		Albuminuria	
	**OR (95%CI)**	***P* value**	**OR (95%CI)**	***P* value**	**OR (95%CI)**	***P* value**	**OR (95%CI)**	***P* value**
**SBP wSD**								
Model 1	1.14 (1.10, 1.18)	<0.001	1.08 (1.05, 1.11)	<0.001	1.09 (1.06, 1.12)	<0.001	1.11 (1.08, 1.14)	<0.001
Model 2	1.09 (1.05, 1.13)	<0.001	1.06 (1.03, 1.09)	<0.001	1.07 (1.04, 1.10)	<0.001	1.10 (1.06, 1.13)	<0.001
Model 3	1.07 (1.03, 1.11)	<0.001	1.04 (1.01, 1.07)	0.029	1.05 (1.02, 1.08)	0.002	1.06 (1.02, 1.09)	0.002
**DBP wSD**								
Model 1	1.11 (1.06, 1.16)	<0.001	1.08 (1.03, 1.12)	0.001	1.07 (1.03, 1.11)	<0.001	1.06 (1.03, 1.10)	0.001
Model 2	1.08 (1.03, 1.13)	0.002	1.06 (1.02, 1.11)	0.007	1.06 (1.02, 1.10)	0.002	1.08 (1.04, 1.13)	<0.001
Model 3	1.07 (1.02, 1.12)	0.005	1.05 (1.01, 1.09)	0.028	1.05 (1.01, 1.09)	0.022	1.05 (1.01, 1.10)	0.025

In model 1, the OR of BPV for target organ damage was adjusted for age, gender, and SBP wSD /DBP wSD

In model 2, the OR of BPV for LVH was additionally adjusted for BMI, cardiovascular disease history, use of antihypertensive drugs, serum creatinine, eGFR, hemoglobin, FBG, serum albumin, and model 1

The OR of BPV for CIMT was additionally adjusted for current smoking, alcohol intake, cardiovascular disease history, FBG, LDL-C, and model 1

The OR of BPV for low eGFR was additionally adjusted for BMI, current smoking, alcohol intake, diabetes mellitus, use of antihypertension drugs, cholesterol, LDL-C, serum albumin, and model 1

The OR of BPV for albuminuria was additionally adjusted for diabetes mellitus, HDL-C, serum albumin, iPTH, blood urea nitrogen, serum calcium, and model 1

In model 3, the OR of BPV for target organ damage was additionally adjusted for hypertension by clinic-SBP/hypertension by clinic-DBP, and model 2

BMI Body mass index, *CIMT* Carotid intima-media thickness, *DBP* Diastolic blood pressure, *GFR* Glomerular filtration rate, *FBG* Blood fasting glucose, *HDL-C* High-density lipoprotein cholesterol, *iPTH* Intact parathyroid hormone, *LDL-C* Low-density lipoprotein cholesterol, *LVH* Left ventricular hypertrophy, *wSD* Weighted standard deviation, *SBP* systolic blood pressure

Supplemental Fig.3 Subgroup analysis

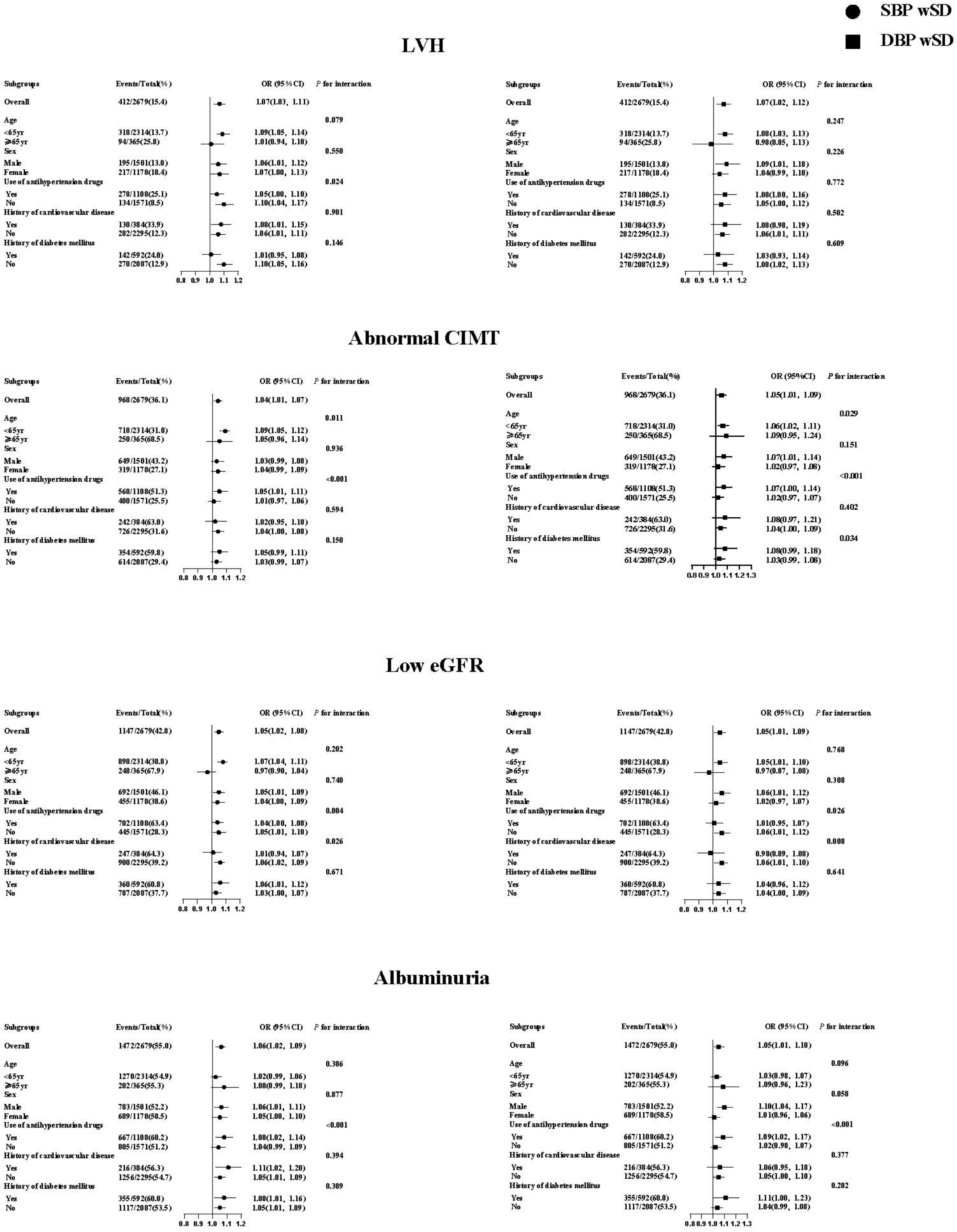

LVH Left ventricular hypertrophy, *CIMT* Carotid intima-media thickness, *GFR* Glomerular filtration rate, *DBP* Diastolic blood pressure, *wSD* Weighted standard deviation; *SBP* Systolic blood pressure
